# Failure of target attainment of beta-lactam antibiotics in critically ill patients and associated risk factors: a two-center prospective study (EXPAT)

**DOI:** 10.1186/s13054-020-03272-z

**Published:** 2020-09-15

**Authors:** Alan Abdulla, Annemieke Dijkstra, Nicole G. M. Hunfeld, Henrik Endeman, Soma Bahmany, Tim M. J. Ewoldt, Anouk E. Muller, Teun van Gelder, Diederik Gommers, Birgit C. P. Koch

**Affiliations:** 1 Department of Hospital Pharmacy, Erasmus University Medical Center, P.O. Box 2040, 3000 CA Rotterdam, the Netherlands; 2 Department of Intensive Care, Maasstad Hospital, Rotterdam, The Netherlands; 3 Department of Intensive Care, Erasmus University Medical Center, Rotterdam, The Netherlands; 4 Department of Medical Microbiology and Infectious Diseases, Erasmus University Medical Center, Rotterdam, The Netherlands; 5 Department of Medical Microbiology, Haaglanden Medical Center, The Hague, The Netherlands; 6 Department of Clinical Pharmacy & Toxicology, Leiden University Medical Center, Leiden, The Netherlands

**Keywords:** Beta-lactam, Critically ill patients, Pharmacokinetics, Pharmacodynamics, Target attainment, Risk factors

## Abstract

**Background:**

Early and appropriate antibiotic dosing is associated with improved clinical outcomes in critically ill patients, yet target attainment remains a challenge. Traditional antibiotic dosing is not suitable in critically ill patients, since these patients undergo physiological alterations that strongly affect antibiotic exposure. For beta-lactam antibiotics, the unbound plasma concentrations above at least one to four times the minimal inhibitory concentration (MIC) for 100% of the dosing interval (100%ƒT > 1–4×MIC) have been proposed as pharmacodynamic targets (PDTs) to maximize bacteriological and clinical responses. The objectives of this study are to describe the PDT attainment in critically ill patients and to identify risk factors for target non-attainment.

**Methods:**

This prospective observational study was performed in two ICUs in the Netherlands. We enrolled adult patients treated with the following beta-lactam antibiotics: amoxicillin (with or without clavulanic acid), cefotaxime, ceftazidime, ceftriaxone, cefuroxime, and meropenem. Based on five samples within a dosing interval at day 2 of therapy, the time unbound concentrations above the epidemiological cut-off (ƒT > MIC_ECOFF_ and ƒT > 4×MIC_ECOFF_) were determined. Secondary endpoints were estimated multivariate binomial and binary logistic regression models, for examining the association of PDT attainment with patient characteristics and clinical outcomes.

**Results:**

A total of 147 patients were included, of whom 63.3% achieved PDT of 100%ƒT > MIC_ECOFF_ and 36.7% achieved 100%ƒT > 4×MIC_ECOFF_. Regression analysis identified male gender, estimated glomerular filtration rate (eGFR) ≥ 90 mL/min/1.73 m^2^, and high body mass index (BMI) as risk factors for target non-attainment. Use of continuous renal replacement therapy (CRRT) and high serum urea significantly increased the probability of target attainment. In addition, we found a significant association between the 100%ƒT > MIC_ECOFF_ target attainment and ICU length of stay (LOS), but no significant correlation was found for the 30-day survival.

**Conclusions:**

Traditional beta-lactam dosing results in low target attainment in the majority of critically ill patients. Male gender, high BMI, and high eGFR were significant risk factors for target non-attainment. These predictors, together with therapeutic drug monitoring, may help ICU clinicians in optimizing beta-lactam dosing in critically ill patients.

**Trial registration:**

Netherlands Trial Registry (EXPAT trial), NTR 5632. Registered on 7 December 2015.

## Key messages

- Less than two-thirds of our study population achieved the 100% ƒT > MIC_ECOFF_ target, and only in one-third the 100% ƒT > 4×MIC_ECOFF_ target was achieved.
- Male gender, high BMI, and eGFR ≥ 90 mL/min/1.73 m^2^ were significantly associated with target non-attainment, while the use of CRRT and high serum urea increased the probability of target attainment.
- For the 100% ƒT > MIC_ECOFF_ regression model, target non-attainment was significantly associated with the clinical outcome ICU length of stay, but no significant association was found for the 30-day survival.

## Introduction

Large multicenter studies have reported antibiotic use in 64 to 71% of patients during their stay in the intensive care unit (ICU) [1, 2]. To adequately prevent and treat severe infections in critically ill patients, it is important that patients are treated with an appropriate dosing regimen of antibiotics [3–5]. However, dose-finding studies typically only include non-ICU populations. Various studies have shown that pathophysiological changes related to critical illness (i.e., altered fluid status, changes in serum albumin concentrations, renal and hepatic dysfunction, systemic inflammatory response syndrome, and microvascular failure) substantially change the pharmacokinetics (PK) and thereby the exposure to antibiotics [6, 7]. Moreover, critically ill patients represent a highly heterogeneous population with a wide distribution of patients’ ages, severities of illness, co-morbidities, source of infections, and outcomes [8]. These challenging conditions make it difficult to achieve optimal exposure in critically ill patients when using standard dosing regimens for beta-lactam antibiotics.

Beta-lactam antibiotics are amongst the most commonly used antibiotics in the ICU setting. These antibiotics exhibit time-dependent bacterial killing. Successful outcome is associated with the percentage of time (T) of the dosing interval in which the unbound (free, ƒ) serum antibiotic concentration remains above the minimum inhibitory concentration (ƒT > MIC). For these antibiotics, the ƒT > MIC value needed for bactericidal activity is between 40 and 70% in in vivo infection models [9, 10], although clinical data suggests optimal efficacy is achieved at 100% ƒT > MIC in critically ill patients [10–12]. Patients achieving 100% ƒT > MIC have significantly higher rates of clinical cure and bacteriological eradication [12–15]. To maximize the probability of clinical efficacy in critically ill patients, unbound plasma concentration from one up to four times the MIC for 100% of the dosing interval (100%ƒT > 1–4×MIC) has been identified as pharmacodynamic targets (PDTs) [16–19]. Further increasing the exposure does not appear to increase the rate or extent of bacterial killing [20].

Target attainment is reported in only 40 to 60% of critically ill patients treated with beta-lactam antibiotics [21, 22]. That said, achieving the high ICU targets is not easy, particularly when conventional beta-lactam dosing regimens are used. Simply increasing the standard dosing for this group of antibiotics in all critically ill patients is not an optimal strategy, since high dosing regimens might result in trough levels associated with overexposure and toxicity [23]. Thus, it appears necessary to individualize beta-lactam dosing regimens in critically ill patients. Accordingly, identifying patients at risk could prompt clinicians to consider more individualized dosing regimens and regular therapeutic drug monitoring (TDM).

To our knowledge, only a few other studies have attempted to quantify patient characteristics as potential predictors for beta-lactam antibiotics target attainment in critically ill patients [14, 24–27]. In some of these studies, limited numbers of patients and/or different beta-lactam antibiotics were investigated, while in only two of these studies, target attainment and relevant factors associated with clinical outcomes were investigated [24, 27]. However, the relationship between target attainment and the clinical outcomes ICU length of stay (LOS) and mortality has not yet been clarified. Therefore, the goals of this study are to determine the prevalence of target attainment of six frequently used beta-lactam antibiotics in ICUs in Europe and to identify risk factors and clinical outcomes associated with target non-attainment.

## Methods

### Study design

This prospective, observational, two-center pharmacokinetics/pharmacodynamics (PK/PD) study was performed in the ICU departments of the Erasmus University Medical Center and Maasstad Hospital, Rotterdam, the Netherlands. The study protocol (EXPAT, NL53551.078.15) was approved by the Erasmus MC Medical Ethics Committee.

### Study population and size

All patients admitted to the ICU between January 2016 and June 2017 and treated for a (presumed) infection with intravenous amoxicillin (with or without clavulanic acid), cefotaxime, ceftazidime, ceftriaxone, cefuroxime, or meropenem were assessed for inclusion. Eligible for enrollment were patients (1) aged ≥ 18 years, (2) expected ICU stay > 72 h, and (3) intravenous intermittent therapy of the study antibiotics. Initiation of study antibiotics, dosage, and duration of therapy were selected during a daily routine multidisciplinary consultation between the attending physician and an infectious disease specialist. Patients were excluded if (1) written informed consent was not obtained, (2) antibiotics were stopped before sampling, or (3) admitted to the ICU for burn injuries. Patient information was collected, including demographic data, clinical data, laboratory data, and antibiotic dosing data during hospitalization within the first 3 days after the start of the antibiotic therapy.

A formal sample size calculation was not needed because of the descriptive and noncomparative setting of this study. A priori in our protocol, it was likely that at least for four of the target antibiotics a minimum of 20 patients could be included based on prescribing data in our study sites. For the analysis of PDT attainment and associated clinical outcomes, a sample size of at least 140 was anticipated to be adequate [28].

### Sample collection and analysis

On day 2 after the start of antimicrobial therapy, in total, five venous blood samples were collected at 15–30 min before the start of a dose (trough concentration, *C*_min_), 15–30 min (peak concentration, *C*_max_), 1 h and 3 h after the end of infusion, and at 15–30 min before the start of the next dose (second *C*_min_). The exact sampling times and the dosage administered were recorded. Blood samples were stored at 2–8 °C directly after drawing to maintain the integrity and centrifuged at 3000 rpm for 6 min within 24 h after collection. The plasma was transferred to cryo-vials for frozen storage (− 80 °C) until analysis. Plasma concentrations were determined by a multi-analyte UPLC-MS/MS [29]. The method was comprehensively validated according to the Food and Drug Administration (FDA) guidance on bioanalytical method validation [30]. All observed concentrations were corrected for protein binding in critically ill patients, using average plasma protein binding (PPB) values [31]. Non-compartmental PK analysis of the plasma concentration–time data was performed using PKSolver (version 2.0) [32].

### Primary endpoints

The PK/PD endpoints were the unbound concentration above the MIC at 100% (ICU target) of the dosing interval (ƒT > MIC and ƒT > 4×MIC). The percentage ƒT > MIC was determined by calculating the intercept of the MIC values with the concentration–time curve. For each of the antibiotics, the epidemiological cut-off (ECOFF) of the presumed pathogens, i.e., the highest MIC for organisms devoid of phenotypically detectable acquired resistance mechanisms, as defined by the European Committee on Antimicrobial Susceptibility Testing (EUCAST), was used [33]. The following EUCAST epidemiological cut-off (MIC_ECOFF_) values were used: amoxicillin 8 mg/L (*Enterobacterales*), cefotaxime 4 mg/L (*Staphylococcus aureus*), ceftazidime 8 mg/L (*Pseudomonas aeruginosa*), ceftriaxone 0.5 mg/L (*Enterobacterales*), cefuroxime 8 mg/L (*Escherichia coli*), and meropenem 2 mg/L (*Pseudomonas aeruginosa*). To assess the suitability of the empirical fixed dosing regimens considering ƒT > MIC and ƒT > 4×MIC, a MIC distribution of 0.03125–128 mg/L was tested for target attainment for each of the antibiotics.

### Secondary endpoints

We defined ICU length of stay (LOS) and 30-day survival from the start of therapy (enrollment) as our secondary endpoints. Factors likely to contribute to these two outcomes were analyzed for association based on clinical relevancy and previously described relationships [14, 24–27]. These included patient characteristics (age, gender, body mass index (BMI)), illness severity score (Sequential Organ Failure Assessment (SOFA) score at the start of target antibiotic), serum albumin, serum urea, sepsis, estimated glomerular filtration rate (eGFR ≥ 90 mL/min/1.73 m^2^), and presence of continuous renal replacement therapy (CRRT).

### Statistical analysis

All statistical analyses were performed using IBM-SPSS (version 24.0, IBM Corp., New York, NY, USA) and R software (version 3.3.3, R Project for Statistical Computing). Normality was assessed using the Shapiro–Wilk test. We analyzed our data using the following three steps. First, categorical variables were expressed as frequencies (percentages), and continuous variables were described as median values with the interquartile range (IQR; 25–75th percentile). Differences in categorical variables were calculated using Pearson chi-square test or Fisher’s exact test as appropriate. The Mann–Whitney *U* test was used to compare continuous variables.

For our primary outcome, i.e., PDT attainment, we estimated multivariate binary logistic regression analyses and present the odds ratios (ORs) and 95% confidence intervals (95% CI). We included SOFA score at inclusion in the multivariate analysis to control for all our regressions for clinically and relevant baseline characteristics.

For our secondary outcomes, we estimated multivariate negative binomial regression and binary logistic regression models examining the association of PDT attainment with ICU LOS and 30-day survival, respectively. For these regressions, we present the ORs and 95% CI. Statistical significance was accepted at *p* ≤ 0.05.

## Results

Overall, a total of 147 patients were included in the study, and 712 serum samples were analyzed. Baseline patient demographic and clinical characteristics, stratified by the target attainment, are summarized in Table 1. The median patient age was 63 years, and 61% of the patients were male. The median ICU LOS was 9 days, and 30-day all-cause mortality rate was 19.7%.

**Table 1:** Baseline demographic characteristics, clinical data, PK/PD indices, and clinical outcomes of all patients included and between PDT attainment and non-attainment groups

Characteristics	All patients (***n*** = 147)	PDT attainment (***n*** = 93)	PDT non-attainment (***n*** = 54)	***p*** value^**a**^
**Demographic data**				
Age (years)	63 (56–70)	65.0 (58.5–73.0)	60.5 (51.0–66.0)	**0.001**
Sex (male/female)	91/56	51/42	40/14	**0.021**
Length (cm)	172.2 (10.7)	169.7 (10.1)	176.6 (10.3)	**< 0.001**
Weight (kg)	77 (70–90)	75 (68–90)	80 (70–90)	0.339
BMI	26.1 (22.9–29.3)	26.9 (23.9–29.6)	25.3 (22.2–28.1)	0.087
Concomitant antibiotics				**0.029**
No	54 (36.7%)	28 (30.1%)	26 (48.1%)	
Yes^b^	93 (63.3%)	65 (69.9%)	28 (51.9%)	
**Clinical data at inclusion**				
SOFA	11.0 [7.0–15.0]			0.293
0–6	28 (19.0%)	15 (16.3%)	13 (24.1%)	
7–9	32 (21.8%)	18 (19.6%)	14 (25.9%)	
10–14	38 (25.9%)	24 (26.1%)	14 (25.9%)	
15	48 (32.7%)	35 (38.0%)	13 (24.1%)	
APACHE II	23 [18–27]			0.161
0–9	5 (3.4%)	3 (3.3%)	2 (3.7%)	
10–19	39 (26.7%)	20 (21.7%)	19 (35.2%)	
20–29	85 (58.2%)	55 (59.8%)	30 (55.6%)	
≥ 30	17 (11.6%)	14 (15.2%)	3 (5.6%)	
Albumin (g/L)	26.3 (7.3)	24.9 (7.0)	28.5 (7.3)	**0.003**
Serum creatinine (μmol/L)	102 [67–155]	124.0 [79.5–182.5]	79.5 [56.8–106.3]	**< 0.001**
Temperature (°C)	36.9 [36.1–37.4]	36.7 [36.0–37.3]	37.0 [36.3–37.6]	0.112
WBC (× 10^9^/L)	13.2 [8.7–18.2]	11.7 [7.3–17.8]	15.8 [10.7–20.2]	**0.022**
CRP (mg/L)	111 [35–226]	120 [46–242]	91 [15–175]	0.072
Serum urea (mmol/L)	8.9 [6.1–16.5]	12.4 [7.1–19.2]	6.6 [4.8–9.3]	**< 0.001**
eGFR (mL/min/1.73 m^2^)				**< 0.001**
< 30	29 (19.7%)	25 (26.9%)	4 (7.4%)	
30–50	31 (21.1%)	28 (30.1%)	3 (5.6%)	
50–90	41 (27.9%)	19 (20.4%)	22 (40.7%)	
> 90	46 (31.3%)	21 (22.6%)	25 (46.3%)	
CRRT				0.063
No	119 (81%)	71 (76.3%)	48 (88.9%)	
Yes	28 (19%)	22 (23.7%)	6 (12.1%)	
**PK/PD indices**				
%ƒT > MIC_ECOFF_	84.2%			
%ƒT > 4×MIC_ECOFF_	51.7%			
**Clinical outcomes**				
ICU LOS (days)	9 [4–15]	11 [6–20]	5 [3–12.8]	**0.005**
30-day mortality	29 (19.7%)	22 (24.2%)	7 (13.2%)	0.135

Values are presented as numbers (%), median [25%–75% interquartile range], or mean (± standard deviation). The numbers in bold are statistically significant

*APACHE II* Acute Physiology and Chronic Health Evaluation II, *BMI* body mass index, *CRP* C-reactive protein, *CRRT* continuous renal replacement therapy, *ECOFF* epidemiological cut-off value, *eGFR* estimated glomerular filtration rate, calculated with the CKD-EPI Creatinine Equation, *ƒT > MIC* the unbound concentrations above the minimum inhibitory concentration, *ICU LOS* intensive care unit length of stay, calculated from the start of study antibiotic until ICU discharge, *PDT* pharmacodynamic target, *SOFA score* Sequential Organ Failure Assessment score, *WBC* white blood cell count

^a^The *p* value between target attainment versus non-attainment patient population and the value in bold indicates a significant difference between the two groups (*p* ≤ 0.05)

^b^One or more additional antibiotics

### Pharmacokinetic parameters

**Fig. 1:**
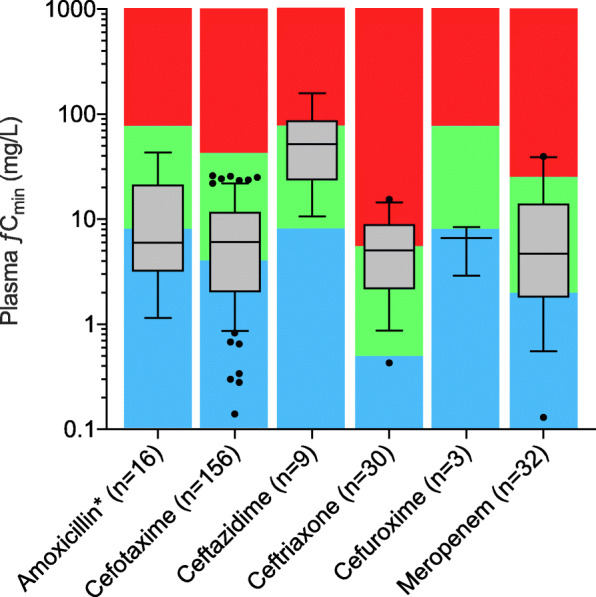
Box (median, 25th and 75th percentiles) and whisker (10th and 90th percentiles) plots of unbound trough (ƒ*C*_min_) plasma concentrations observed in critically ill patients treated with beta-lactam antibiotics. The green areas indicate the target exposure (ƒ*C*_min_ = 1–10×MIC_ECOFF_), the blue areas indicate suboptimal exposure (ƒ*C*_min_ <1×MIC_ECOFF_), and the red areas indicate threshold for dose reduction (ƒ*C*_min_ > 10×MIC_ECOFF_). The numbers of trough samples (*n*) are presented per antibiotic. Outliers are removed using the ROUT method (*Q* = 0.5%). Filled circles are remaining outliers. *Amoxicillin and amoxicillin/clavulanic acid

Box-and-whisker plots of unbound trough (ƒ*C*_min_) plasma concentrations observed for the different antibiotics are shown in Fig. 1, and peak (ƒ*C*_max_) plasma concentration plots can be found in Additional file 1. Large inter-patient variability was observed in the plasma concentration of the various antibiotics, e.g., a ƒ*C*_min_ of cefotaxime ranging from 0.14 to 26 mg/L. Detailed data describing the pooled antibiotic dosing and PK/PD indices are provided in Additional file 2.

### Target attainment

**Fig. 2:**
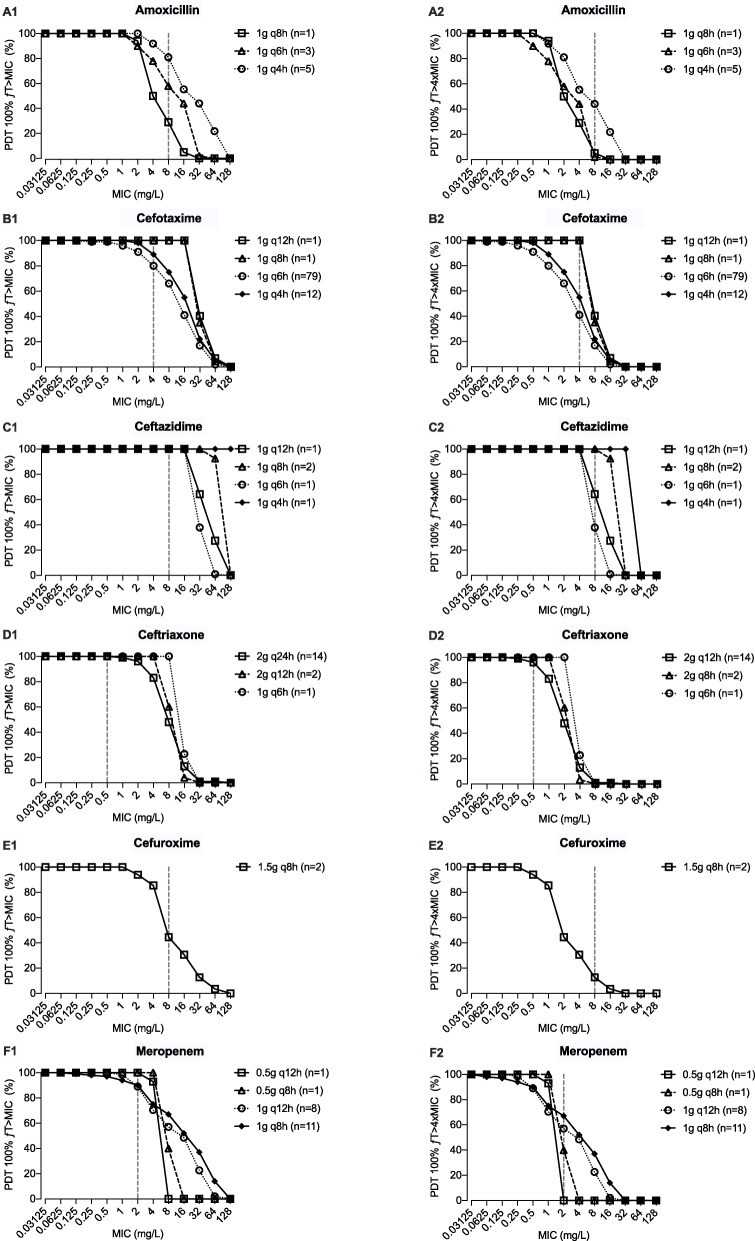
Target attainment in ICU patients for various beta-lactams and dosing regimens to reach the PDTs 100% ƒT > MIC (**A1**–**F1**) and 100% ƒT > 4×MIC (**A2**–**F2**) for a range of MICs. The numbers of patients (*n*) are presented per antibiotic and dose regimen. The dotted horizontal line indicates the intercept with the EUCAST epidemiological cut-off (ECOFF) breakpoints: amoxicillin 8 mg/L (*Enterobacterales*), cefotaxime 4 mg/L (*Staphylococcus aureus*), ceftazidime 8 mg/L (*Pseudomonas aeruginosa*), ceftriaxone 0.5 mg/L (*Enterobacterales*), cefuroxime 8 mg/L (*Escherichia coli*), and meropenem 2 mg/L (*Pseudomonas aeruginosa*)

The proportions of patients achieving the PDT of 100% ƒT > MIC_ECOFF_ and 100% ƒT > 4×MIC_ECOFF_ were 63.3% (93/147) and 36.7% (44/147), respectively (Table 1 and Additional file 2). Compared to patients who did attain the target, younger male patients with higher creatinine clearance, higher serum albumin, higher white blood cell count, higher length, and lower urea and those who received concomitant antibiotics were more likely not to achieve the PDT. Moreover, 45.7% (21/46) and 31% (16/51) of those with an eGFR ≥ 90 mL/min /1.73 m^2^ at baseline and day 2 achieved the 100% ƒT > MIC_ECOFF_ target, respectively. Of all patients, twenty-eight (19%) patients were treated with CRRT. Although not statistically significant, there was a noticeable clinically significant difference in the number of patients treated with CRRT between the groups who did and did not achieve the PDT, respectively 23.7% and 12.1% (*p* = 0.06). The rates for 100% ƒT > MIC_ECOFF_ target attainment for amoxicillin, cefotaxime, ceftazidime, ceftriaxone, cefuroxime, and meropenem were 44.4%, 57.0%, 100%, 94.1%, 0%, and 71.4%, respectively (Additional file 2). Probability of reaching the target 100%ƒT > 4×MIC_ECOFF_ was less than 25% for amoxicillin, cefotaxime, and cefuroxime, suggesting that for all these drugs, inadequate drug concentrations are obtained for pathogens with high MICs. Target attainment for various beta-lactam antibiotics and dosing regimens to reach the PDTs of 100% ƒT > MIC and 100% ƒT > 4×MIC for a range of MICs (0.03125 to 128 mg/L) are shown in Fig. 2.

### Predictors for target (non-)attainment

Predictor variables for target attainment were investigated in the multivariate binary logistic regression (Table 2). Variables associated with 100% ƒT > MIC_ECOFF_ target attainment include treatment with CRRT (OR 6.54, 95% CI 1.47–48.61) and high serum urea (OR 1.09, 95% CI 1.03–1.17). Male gender, on the other hand, was found to be significantly correlated with target non-attainment (OR 0.32, 95% CI 0.12–0.81). For the PDT 100% ƒT > 4×MIC_ECOFF_, target non-attainment was significantly more observed in patients with baseline eGFR ≥ 90 mL/min/1.73 m^2^ (OR 0.14, 95% CI 0.03–0.49) and high BMI (OR 0.91, 95% CI 0.83–0.99). A high serum urea demonstrated a significant association (OR 1.05, 95% CI 1.00–1.10) with 100% ƒT > 4×MIC target attainment.

**Table 2:** Multivariate binary logistic regression in ICU patients, analysis predicting attainment achieving PDT of (A) 100% ƒT > MIC and (B) 100% ƒT > 4×MIC as the dependent factor

Predictor variables	100% ƒT > MIC	100% ƒT > 4×MIC
	OR (95% CI)	OR (95% CI)
Male gender	**0.32 (0.12–0.81)**	0.60 (0.23–1.51)
Age (years)	1.03 (0.99–1.07)	0.98 (0.94–1.01)
BMI (mg/kg^2^)	0.98 (0.91–1.05)	**0.91 (0.83–0.99)**
Serum urea (mmol/L)	**1.09 (1.03–1.17)**	**1.05 (1.00–1.10)**
eGFR ≥ 90 (mL/min/1.73 m^2^)	0.69 (0.25–1.94)	**0.14 (0.03–0.49)**
SOFA score	1.05 (0.96–1.16)	0.95 (0.85–1.05)
CRRT	**6.54 (1.47–48.61)**	2.26 (0.73–6.97)
Sepsis	1.18 (0.38–3.88)	1.31 (0.43–3.88)

The estimates are odds ratios (ORs) and 95% confidence intervals. The numbers in bold are statistically significant. Statistical significance was accepted at *p* ≤ 0.05. McFadden *R*-squared for models A and B are 0.21 and 0.18, respectively, representing good fit

*BMI* body mass index, *CRRT* continuous renal replacement therapy, *eGFR* estimated glomerular filtration rate calculated with the CKD-EPI Creatinine Equation, *ƒT > MIC* the unbound concentrations above the minimum inhibitory concentration, *PDT* pharmacodynamic target, *SOFA score* Sequential Organ Failure Assessment score

### Predictors for clinical outcomes

Table 3 shows the estimates for the multivariate binomial regression and binary logistic regression models examining the association of target attainment with ICU LOS and 30-day survival, respectively. The PDT of 100% ƒT > MIC_ECOFF_ was significantly associated with the clinical outcome ICU LOS (OR 1.66, 95% CI 1.19–2.32). In the multivariate models, the presence of CRRT was for both PDTs significantly associated with higher ICU LOS (OR, 2.08 [95% CI, 1.38–3.20] and OR, 2.13 [95% CI, 1.39–3.34], respectively). Furthermore, an eGFR ≥ 90 mL/min/1.73 m^2^ was associated with increased ICU LOS in both PDT models (OR, 1.67 [95% CI, 1.13–2.47] and OR, 1.69 [95% CI, 1.12–2.56], respectively). Finally, there was no significant association for both models with the 30-day survival outcome (OR, 0.56 [95% CI, 0.19–1.66] and OR, 1.24 [95% CI, 0.44–3.73], respectively).

**Table 3:** Multivariate regression models in ICU patients for PDT attainment and odds ratio estimates for the association with the clinical outcomes (A) ICU LOS and (B) 30-day survival

Models and variables	ICU LOS^**a**^ OR (95% CI)	30-day survival^**b**^ OR (95% CI)
**Regression model, PDT: 100% ƒT > MIC**	**1.66 (1.19–2.32)**	0.58 (0.19–1.66)
Age (years)	0.99 (0.98–1.01)	1.02 (0.98–1.05)
CRRT	**2.08 (1.38–3.20)**	0.41 (0.13–1.33)
Sepsis	0.91 (0.61–1.37)	0.90 (0.30–2.86)
Serum urea (mmol/L)	1.00 (0.99–1.02)	1.05 (0.99–1.11)
SOFA score	1.00 (0.96–1.03)	0.95 (0.85–1.05)
eGFR ≥ 90 (mL/min/1.73 m^2^)	**1.67 (1.13–2.47)**	1.97 (0.66–6.88)
**Regression model, PDT: 100% ƒT > 4**×**MIC**	1.26 (0.88–1.82)	1.24 (0.44–3.73)
Age (years)	1.00 (0.98–1.01)	1.01 (0.98–1.05)
CRRT	**2.13 (1.39–3.34)**	0.35 (0.11–1.11)
Sepsis	0.89 (0.59–1.34)	0.91 (0.31–2.88)
Serum urea (mmol/L)	1.01 (0.99–1.03**)**	1.04 (0.98–1.10)
SOFA score	1.01 (0.97–1.05)	0.94 (0.84–1.05)
eGFR ≥ 90 (mL/min/1.73 m^2^)	**1.69 (1.12–2.56)**	2.09 (0.66–7.22)

The estimates are odds ratios (ORs) and 95% confidence intervals. The numbers in bold are statistically significant. Statistical significance was accepted at *p* ≤ 0.05

*CRRT* continuous renal replacement therapy, *eGFR* estimated glomerular filtration rate, *ICU LOS* intensive care unit length of stay, calculated from the start of study antibiotic until ICU discharge, *PDT* pharmacodynamic target, *SOFA* Sequential Organ Failure Assessment

^a^Negative binomial regression model

^b^Binary logistic regression model

## Discussion

In this prospective study, we describe detailed target attainment of six frequently used beta-lactam antibiotics and risk factors for target non-attainment in critically ill patients. Achievement of PK/PD targets was highly variable in the beta-lactam antibiotics analyzed in this study (Fig. 1), and the 100% ƒT > MIC_ECOFF_ target was achieved in 63.3% of the study population. We identified male gender, high BMI, and high eGFR as risk target non-attainment in our study population. Moreover, we found a significant association between the 100%ƒT > MIC_ECOFF_ target attainment and ICU LOS, but no significant correlation was found for the 30-day survival.

Roberts et al. found that critically ill patients failing to attain even the most conservative beta-lactam exposure target of 50% ƒT > MIC were 32% less likely to have a positive clinical outcome (defined as completion of treatment course without change or addition of antibiotic therapy) [21]. In addition, they found an association between positive clinical outcome and an increasing 100% ƒT > MIC ratio (OR 1.53, *p* = 0.03) [21]. Considering that critically ill patients are vulnerable to suboptimal dosing and represent a source of selection of resistance to antibiotics, we also assessed the probability of 100% ƒT > 4×MIC_ECOFF_ target attainment, as this would allow for the maximal bacterial killing and also protect against bacterial regrowth [34–36]. In our study, the 100% ƒT > 4×MIC_ECOFF_ target was achieved in only 36.7% of the patients.

Antimicrobial dosing in critically ill patients requires consideration of drug distribution and clearance in the setting of end-organ failure, fluctuations in fluid status, and drug interactions. However, the findings of our study suggest that target attainment during beta-lactam therapy in critically ill patients may be anticipated at the bedside prior to antibiotic initiation. Predictor variables in the multivariate analysis associated with increased odds for target non-attainment were male gender and eGFR (Table 2). These associations in critically ill patients treated with beta-lactam antibiotics are in line with previous studies [25–27].

The observed effect of gender on drug exposure can be explained by the fact that, on average, greater volume of distribution (plasma volume and intra-/extracellular water) and higher drug clearance is observed in men [37]. Although gender is easy to implement in risk factor models for target non-attainment, future studies should be designed with a primary focus on this topic to better understand of the basic mechanisms of gender differences and the implications for clinical management [37].

In our study population, the probability of the 100%ƒT>4×MIC target non-attainment was significantly associated with an eGFR ≥ 90 mL/min/1.73 m^2^ (OR 0.14, 95% CI 0.03–0.49) at inclusion. eGFR calculated on serum creatinine is the best surrogate marker of renal clearance. In total, 31.3% of our population had an eGFR ≥ 90 mL/min/1.73 m^2^, while 46.3% in the non-attainment group did so (Table 2). This means that patients with presumed “normal” or elevated renal function are at risk of target non-attainment and need to be identified early so that appropriate dose adjustment can be made. Moreover, Imani et al. assessed the performance of eGFR as an independent predictor for target non-attainment using a ROC curve and found an eGFR threshold value of ≥ 71.5 mL/min/1.73 m^2^ had a sensitivity and specificity of 77% and 65%, respectively [26]. As beta-lactam antibiotics are predominantly cleared by the kidney, high renal function (eGFR ≥ 130 mL/min), as observed in augmented renal clearance (ARC), contributes even more to suboptimal PK/PD target attainment. ARC is observed in 30–65% of patients during the first week in the ICU [14, 38, 39], and both age and male gender are independently associated with ARC [39]. Moreover, ARC is a strong predictor for one or more undetectable trough concentrations [OR 3.3, 95% CI 1.11–9.94] [14]. Carrié et al. reported that eGFR ≥ 170 mL/min were significantly associated with T<4×MIC [OR 10.1; (2.4–41.6); *p* = 0.001] [24]. The ability to rapidly predict the risk of target non-attainment in patients with ARC using available eGFR has considerable clinical value.

Furthermore, in the multivariate analysis, we found a strong and significant association between target attainment and treatment with CRRT. To our knowledge, this is the only study where in a multivariate analysis this association was demonstrated. Not surprisingly, considering that beta-lactams are predominantly cleared via renal elimination. At the same time, these patients may be at risk for overexposure and toxicity due to the reduced elimination. However, predicting beta-lactam concentrations during treatment with CRRT is challenging, as both volume of distribution and total drug clearance are affected, and both parameters may be significantly disturbed during critical illness. In addition, it is important to realize that the effect of CRRT on target attainment may be unpredictably affected by for example the type of membrane, device settings, and intensity [40].

Evidence regarding relevant predictor variables with clinical outcomes in critically ill patients are still limited. Huttner et al. found that ARC was associated with undetectable beta-lactam antibiotic trough concentrations, but failed to demonstrate a link between ARC or low beta-lactam trough concentrations with clinical failure [14]. In contrast, Carrié et al. reported that beta-lactam underexposure was associated with higher rates of therapeutic failure in septic critically ill patients [24]. In our population, we found a significant association for the 100% ƒT > MIC_ECOFF_ PDT model with the clinical outcome ICU LOS. The link with this outcome is a new finding. Interestingly, the ICU LOS increases as 100% ƒT > MIC is achieved. This is also reflected in the significant difference of the median admission days in both groups (Table 1). An explanation for the target attainment association with ICU LOS may be the fact that the elderly and the most ill patients in our population stayed longer at the ICU. These patients have relatively worse end-organ functions (including renal clearance) and therefore have higher exposure and are more on-target. Patients who died could have had an effect on the outcomes of the predictive LOS models. Therefore, we also performed the binomial regression models while excluding the non-survivors (data not presented). However, this had no effect on the significant variables found in both PDT models. Furthermore, in both PDT models, CRRT was significantly associated with higher ICU LOS. In addition, eGFR ≥ 90 mL/min/1.73 m^2^ was independently and convincingly associated with increased ICU LOS in both PDT models (Table 3). It should be noted that patients receiving CRRT (*n* = 28) at any time during the antibiotic therapy were not excluded from this analysis. However, an eGFR ≥ 90 mL/min/1.73 m^2^ was still independently associated with increased ICU LOS when patients on CRRT are excluded (data not shown). Although increased renal clearance and ARC is highly prevalent in critically ill patients, in practice, clinicians may fail to address this as a risk factor and prescribe standard beta-lactam dosing. Indeed, the complexity and dynamic nature of critically ill patients and the heterogeneity in their pharmacokinetic response make associations of clinical variables and the calculated risk of target non-attainment of beta-lactam antibiotics difficult to apply without supporting tools.

Therapeutic drug monitoring (TDM) combined with population PK models with appropriate co-variables can be used to interpret the complex PK in critically ill patients and to support in optimizing individual dosing to improve predefined targets [41–43]. However, the lack of guidelines, long turnaround times, and limited access to beta-lactam TDM services are potential barriers to its implementation [44]. Routine TDM with same-day antibiotic dose adaptation using immunoassays over the more commonly used chromatographic methods could contribute to overcoming these barriers in routine clinical practice [45]. However, pending a large randomized trial investigating the effect of TDM of beta-lactam antibiotics on clinical outcome in critically ill patients [46], the clinical impact on patient’s prognosis using this strategy is not yet fully demonstrated. In view of the fact that target attainment is observed in only about 60–65% of critically ill patients receiving antibiotics and considering the increasing resistance to antibiotics worldwide, higher dosing in these patients could be an alternative strategy to obtain better target attainment when TDM is not available. Carrie et al. showed that, in critically ill patients with ARC, higher than licensed dosing regimens of beta-lactam antibiotics may be safe and effective in reducing the rate of therapeutic failure [47]. Moreover, Imani et al. found that prescribed daily antibiotic dose ≥ 1.5 times the product information recommendations was associated with better target attainment [26]. Toxicity concerns as a result of drug accumulation are valid, but less pertinent given that toxicity thresholds are high for beta-lactam antibiotic agents [23]. However, serious adverse drug reactions related to excessively high serum levels have recently been reported, which further underscores a potential added value of TDM in critically ill patients [48–51]. Furthermore, to avoid high (peak) serum levels of beta-lactam antibiotics, prolonged or continuous infusion is an alternative dosing strategy to maximize target achievement and is likely to improve clinical outcomes in critically ill patients [52].

The current study has some limitations that should be noted. First, we have measured total drug concentrations with correction for protein binding based on the literature. Measuring unbound concentrations is desirable in critically ill patients, since the ratio of bound and unbound drugs can be subjected to changes because of disease characteristics of these patients. However, with the exception of ceftriaxone, the protein binding of most antibiotics in this study is too low to be clinically affected by, for example, a decreased serum albumin. Moreover, we analyzed unbound concentration of ceftriaxone in another cohort of critically ill patients [46] to support the clinical feasibility of calculating unbound fractions using an average PPB value. The mean fraction of ceftriaxone unbound plasma concentrations (*n* = 34) in the range of 0.05–40 mg/L was 12.3% [IQR 8.5–20] (unpublished observations), which is comparable to the calculated unbound concentration used in this study and previously published data [31]. Second, MIC values were assumed from population estimates (ECOFF values) to calculate target attainment. Due to this approach, there is a chance that target attainment is underestimated in our study. Furthermore, the use of a measured MIC obtained by a single MIC determination is debatable, since routine clinical laboratories cannot determine MICs with sufficient accuracy due to the inherent assay variation in the MIC test and the variation in any MIC determination [53]. Although the ECOFF is in many situations similar to the clinical breakpoint, it is still important to closely evaluate the PK/PD target against the local drug resistance epidemiology. Third, we were not able to perform direct urinary creatinine measurements based on 24-h urine collections. We estimated serum creatinine clearance using the CKD-EPI Creatinine Equation formula, which is not validated for critically ill patients. However, it is unlikely that the study population with an eGFR ≥ 90 mL/min/1.73 m^2^ would in fact have had impaired renal function if calculated with urinary creatinine. Thus, we assume that possible misclassification of creatinine clearance did not invalidate our main observations. Fourth, we are aware that, in our regression models, baseline characteristics and target attainment were measured at two time points in the study period, respectively at inclusion and day of sampling. However, it is important to include some control variables that reflect the baseline characteristics of the patients. Finally, in the study population, cephalosporins, in particular cefotaxime, were overrepresented compared with the other classes of beta-lactam antibiotics. Target attainment and associated risk factors of the different antimicrobial agents in this context thus remain to be determined by more specific studies.

## Conclusions

This study provides additional PDT attainment data and risk factors associated with target non-attainment to support beta-lactam antibiotic dosing in critically ill patients. Traditional beta-lactam antibiotic dosing results in low target attainment, less than two-thirds of our study population achieved the 100% ƒT > MIC_ECOFF_ target and only one-third the 100% ƒT>4×MIC_ECOFF_ target. Target attainment during beta-lactam therapy in critically ill patients may be anticipated at the bedside using predictor variables. Male patients with apparently normal or increased renal function and the use of CRRT were strong predictors of beta-lactam antibiotic exposure. Our data suggest that patients with eGFR ≥ 90 mL/min/1.73 m^2^ are at risk of target non-attainment, and this is associated with increased ICU LOS. Based on our data, we recommend TDM, selectively applied based on the described risk factors, during the early stages of beta-lactam therapy in critically ill patients. Whether these patients would benefit from more individualized dosing regimens should be evaluated by randomized controlled studies.

## Supplementary information

**Additional file 1: Figure S1.** Box and whisker plots of unbound peak plasma concentrations observed in critically ill patients treated with six beta-lactam antibiotics.

**Additional file 2: Table S1.** Characteristics of pooled antibiotic dosing and pharmacokinetic/pharmacodynamic (PK/PD) data.

## Abbreviations

APACHE: Acute Physiology and Chronic Health Evaluation
ARC: Augmented renal clearance
CL: Clearance
*C*_max_: Maximum concentration
*C*_min_: Minimum concentration
CrCl: Creatinine clearance
CI: Continuous infusion
CRP: C-reactive protein
CRRT: Continuous renal replacement therapy
ECOFF: Epidemiologic cut-off values
eGFR: Estimated glomerular filtration rate
EUCAST: European Committee on Antimicrobial Susceptibility Testing
EI: Extended infusion
IM: Intermittent
ICU: Intensive care unit
IQR: Interquartile range
LOS: Length of stay
MIC: Minimal inhibitory concentration
PD: Pharmacodynamic
PDT: Pharmacodynamic target
PI: Product information
PK: Pharmacokinetic
PK/PD: Pharmacokinetic/pharmacodynamic
PPB: Plasma protein binding
SOFA: Sequential Organ Failure Assessment
TDM: Therapeutic drug monitoring
ƒT > MIC: Time for which the unbound drug concentration remains above the minimum inhibitory concentration during a dosing interval
UPLC-MS/MS: Ultra-performance liquid chromatography/tandem mass spectrometry
Vd: Volume of distribution
