# Supplementary material for: Failure of target attainment of beta-lactam antibiotics in critically ill patients and associated risk factors: a two-center prospective study (EXPAT)
Source: Crit Care. 2020 Sep 15;24:558. doi: 10.1186/s13054-020-03272-z (PMC7493358; doi:10.1186/s13054-020-03272-z)
Supplement: Supplementary file 1 — Additional file 1: Figure S1. Box and whisker plots of unbound peak plasma concentrations observed in critically ill patients treated with six beta-lactam antibiotics. [file 13054_2020_3272_MOESM1_ESM.pdf]

SUPPLEMENTAL MATERIAL

Abdulla et al. 2020: Failure of target attainment of beta-lactam antibiotics in critically ill patients and associated risk factors: a two-center prospective study (EXPAT study)

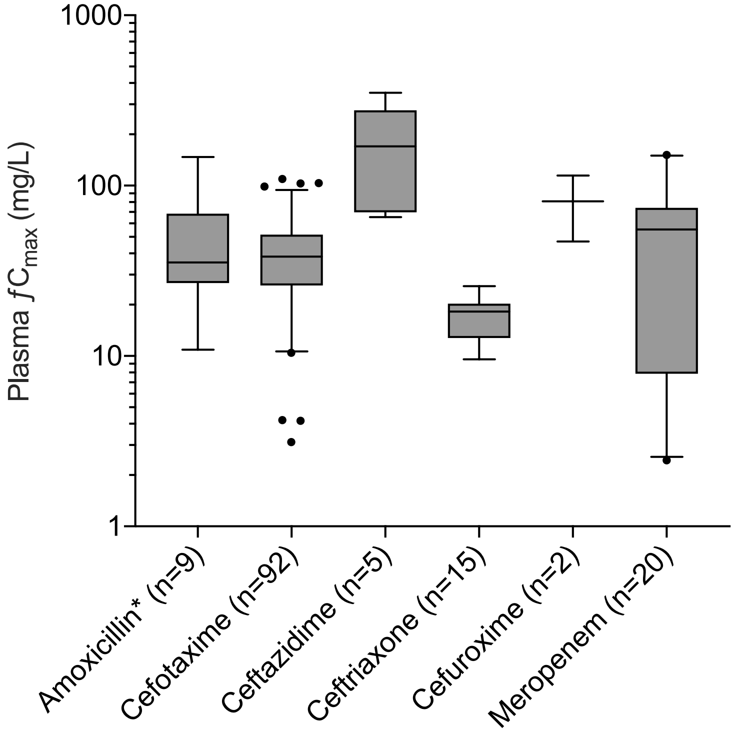

Figure S1. Box (median, 25th and 75th percentiles) and whisker (10th and 90th percentiles) plots of unbound peak (fC_{max}) plasma concentrations observed in critically ill patients treated with six beta-lactam antibiotics. The numbers of peak samples (n) is presented per antibiotic.

Outliers are removed using ROUT method (Q = 0.5%). Filled circles are remaining outliers.

*Amoxicillin and Amoxicillin/Clavulanic acid
