# Supplementary material for: Failure of target attainment of beta-lactam antibiotics in critically ill patients and associated risk factors: a two-center prospective study (EXPAT)
Source: Crit Care. 2020 Sep 15;24:558. doi: 10.1186/s13054-020-03272-z (PMC7493358; doi:10.1186/s13054-020-03272-z)
Supplement: Supplementary file 2 — Additional file 2: Table S1. Characteristics of pooled antibiotic dosing and pharmacokinetic/pharmacodynamic (PK/PD) data. [file 13054_2020_3272_MOESM2_ESM.pdf]

SUPPLEMENTAL MATERIAL
Abdulla et al. 2020: Failure of target attainment of beta-lactam antibiotics in critically ill patients and associated risk factors: a two-center prospective study (EXPAT study)

Table S1. Characteristics of pooled antibiotic dosing and pharmacokinetic/pharmacodynamic (PK/PD) data

**Dosing and PK/PD data**	**Amoxicillin* (n=9)**	**Cefotaxime (n=93)**	**Ceftazidime (n=5)**	**Ceftriaxone (n=17)**	**Cefuroxime (n=2)**	**Meropenem (n=21)**	**Total (n=147)**
Dosage per 24h (g)	6.0 [4.0-6.0]	4.0 [4.0-4.0]	3.0 [2.5-5.0]	2.0 [2.0-2.0]	4.5 [4.5-4.5]	3.0 [2.0-3.0]	
DDD	1.66 ±0.42	1.06 ±0.18	1.20 ±0.45	1.18 ±0.39	1.50	0.83 ±0.21	
No. of samples	4.89 ±0.33	4.94 ±0.25	4.80 ±0.45	4.65 ±0.61	4.50 ±0.71	4.67 ±0.80	4.85 ±0.44
Mean# C_{min} (mg/L)	12 ±13	7.8 ±6.6	60 ±48	5.9 ±4.1	6.0 ±2.8	9.1 ±11	
Median# C_{min} (mg/L)	6.0 [3.2-21]	6.1 [2.0-12]	52 [24-88]	5.1 [2.2-9.0]	6.6 [2.9-8.4]	4.7 [1.8-14]	
Range# C_{min} (mg/L)	1.2-43	0.14-26	11-158	0.43-15	2.9-8.4	0.13-40	
Mean# C_{max} (mg/L)	51 ± 41	42 ±23	173 ±116	17 ±4.7	5.5 ±3.6	53 ±45	
Median# C_{max} (mg/L)	35 [27-51]	38 [26-51]	170 [70-278]	18 [13-20]	5.1 [2.3-8.3]	7.9 [7.9-74]	
Range# C_{max} (mg/L)	11-136	3.1-106	65-286	9.6-16	1.2-11	2.4-149	
%fT>MIC_{ECOFF}	69.8%	81.4%	100%	99.7%	44.3%	90.5%	84.2%
%fT>4xMIC_{ECOFF}	25.6%	46.6%	80.4%	98.3%	12.7%	58.8%	53.7%
40%fT>MIC_{ECOFF}	77.8%	91.4%	100%	100%	50%	95.2%	91.8%
50%fT>MIC_{ECOFF}	66.7%	81.7%	100%	100%	50%	95.2%	85.0%
60%fT>MIC_{ECOFF}	55.6%	75.3%	100%	100%	0%	90.5%	78.9%
70%fT>MIC_{ECOFF}	55.6%	69.9%	100%	100%	0%	90.5%	75.5%
100%fT>MIC_{ECOFF}	44.4%	57.0%	100%	94.1	0%	71.4%	63.3%
100%fT>4xMIC_{ECOFF}	22.2%	17.2%	60%	82.4%	0%	42.9%	36.7%

Values are presented as numbers (%), median [25%-75% interquartile range], or mean (± standard deviation).

* Amoxicillin (n=7) and amoxicillin/clavulanic acid (n=2).

# Outliers removed using ROUT method (Q = 0.5%).

Abbreviations: **DDD**: Defined Daily Dose; **ECOFF**: epidemiological cut-off breakpoint; **$ƒC_{max}$**: unbound peak plasma concentrations; **$ƒC_{min}$**: unbound trough plasma concentration; **ƒT>MIC:** the unbound concentrations above the minimum inhibitory concentration; **IQR**: interquartile range; **MIC**: Minimal Inhibitory Concentration
